# Thymoquinone Is Effective in Painful Paclitaxel-Induced Peripheral Neuropathy Without Compromising Anticancer Activity

**DOI:** 10.3390/biom16091282

**Published:** 2026-09-04

**Authors:** Ibtihal Segmani, Chiara D’Aprile, Laura Cherchi, Eleonora Pozzi, Alessia Chiorazzi, Annalisa Canta, Paola Alberti, Cristina Meregalli, Lisa Fantoni, Elisa Ballarini, Virginia Rodriguez Menendez, Elisabetta Donzelli, Silvia Fermi, Arianna Scuteri, Houda Filali, Guido Cavaletti, Valentina Alda Carozzi

**Affiliations:** 1 Laboratory of Integrated Cancer Research, Faculty of Medicine and Pharmacy, Hassan II University of Casablanca, Casablanca 21100, Morocco; filalipharmaco@gmail.com; 2 Experimental Neurology Unit, School of Medicine and Surgery, University of Milano-Bicocca, 20900 Monz, Italy; chiara.daprile@unimib.it (C.D.); laura.cherchi@unimib.it (L.C.); eleonora.pozzi@unimib.it (E.P.); alessia.chiorazzi@unimib.it (A.C.); annalisa.canta@unimib.it (A.C.); paola.alberti@unimib.it (P.A.); cristina.meregalli@unimib.it (C.M.); lisa.fantoni@unimib.it (L.F.); elisa.ballarini@unimib.it (E.B.); virginia.rodriguez1@unimib.it (V.R.M.); elisabetta.donzelli@unimib.it (E.D.); s.fermi2@campus.unimib.it (S.F.); arianna.scuteri@unimib.it (A.S.); guido.cavaletti@unimib.it (G.C.)

**Keywords:** Thymoquinone, paclitaxel, peripheral neuropathy, *in vivo/in vitro* models, SIRT1 expression

## Abstract

Chemotherapy-Induced Peripheral Neuropathy (CIPN) is a dose-limiting complication of paclitaxel (PTX) therapy, for which effective treatments are still lacking. This study evaluated the neuroprotective potential of Thymoquinone (TQ), a bioactive derivative of *Nigella sativa*, in mitigating chronic PTX-induced neurotoxicity without compromising antineoplastic activity. We first performed *in vitro* experiments using Sprague–Dawley rat embryonic (E15) Dorsal Root Ganglia (DRG) (Envigo Laboratory (Udine, Italy)) to assess neurotoxicity through neurite outgrowth evaluation. To investigate the molecular mechanisms underlying TQ’s putative neuroprotective mechanisms, SIRT1 protein expression was additionally evaluated by western blot, while MCF-7 and MDA-MB-231 breast cancer cells were used to monitor cytotoxicity via MTT assay. We then moved to *in vivo* experiments, in which chronic neuropathy was induced in rats using PTX (10 mg/kg, i.v., weekly for 4 weeks). TQ was co-administered orally (5–10 mg/kg/day). The effects of TQ on peripheral neuropathy were assessed through behavioral testing, neurophysiological assessments, and histological analysis of Intraepidermal Nerve Fibre density (IENF), DRG and peripheral nerves. *In vitro*, TQ (5 μM) significantly attenuated PTX-induced neurite shortening at 24 h; TQ co-treatment fully prevented PTX-induced SIRT1 downregulation in embryonic DRG neurons and did not compromise PTX cytotoxicity in MCF-7 cells, while significantly potentiating it in MDA-MB-231 triple-negative breast cancer cells. *In vivo* results demonstrated that PTX-treated animals exhibited mild erythroid myelosuppression at the end of treatment. Regarding efficacy, TQ consistently prevented PTX-induced mechanical allodynia throughout the treatment period, and TQ (10 mg/kg) transiently mitigated IENF depletion at mid-treatment; however, no improvement in neurophysiological parameters or peripheral nerve morphology was observed at either time point. Collectively, these findings suggest that, under conditions of chronic PTX exposure, TQ exerts a predominantly analgesic effect *in vivo*, without conferring meaningful structural neuroprotection against PTX-induced peripheral nerve degeneration.

## 1. Introduction

Chemotherapy remains the predominant modality for cancer treatment despite the development of new therapies, including immunotherapy. Nevertheless, it is associated with a range of potentially life-threatening side effects [1,2]. Among these complications, Chemotherapy-Induced Peripheral Neuropathy (CIPN) represents a prevalent and debilitating toxicity associated with a wide range of cytotoxic agents, including taxanes, platinum compounds, proteasome inhibitors, and vinca alkaloids [3,4]. Importantly, incidence, severity, and clinical manifestation of CIPN vary according to the chemotherapeutic agent used, cumulative dose, and treatment regimen, reflecting differences in the underlying mechanisms of neurotoxicity [5,6].

Among the antineoplastic drugs associated with CIPN, Paclitaxel (PTX), a plant-derived taxane, is widely used as a first-line treatment for various solid tumors, including breast, prostate, ovarian, and non-small cell lung cancers [7]. Despite its high therapeutic index against malignancies, PTX is associated with a pronounced incidence of peripheral neuropathy (named Paclitaxel-Induced Peripheral Neuropathy, PIPN), affecting approximately 60–70% of patients [7,8]. This neurotoxicity arises not only from microtubule stabilization but also from mitochondrial dysfunction and ROS-mediated oxidative stress, leading to neuroinflammatory pathway activation and consequent persistent neuronal injury. As a result, PIPN typically presents with sensory disturbances, including paresthesia and dysesthesia, with a “stocking-and-glove” distribution, and neuropathic pain, which significantly impairs quality of life and often necessitates dose reductions or premature discontinuation of life-saving therapies [9,10,11].

The clinical management of PIPN presents a considerable challenge, as existing pharmacological and non-pharmacological interventions frequently demonstrate limited efficacy [12,13]. To date, only the antidepressant Duloxetine is moderately recommended by the American Society of Clinical Oncology (ASCO) for PIPN prevention [14]. However, since this solution applies only to the symptomatic treatment of CIPN, much work is still needed to address a neuroprotective strategy.

To effectively counteract painful PIPN, research has increasingly focused on understanding key molecular pathways and identifying potential therapeutic targets. Among these, Silent Information Regulator 1 (SIRT1), a NAD-dependent histone deacetylase, has emerged as a promising neuroprotective target involved in neuronal survival, axonal integrity, and nociceptive signaling [15]. Numerous researchers have demonstrated that SIRT1 provides neuroprotection mainly by inhibiting neuroinflammation mediated by NF-κB and reducing oxidative stress [16]. Importantly, SIRT1 downregulation has been shown to impair neurite outgrowth and neuronal viability in sensory neurons, and its activation has been associated with alleviation of neuropathic pain in experimental models, including those associated with chemotherapeutic agents, making it a particularly relevant mechanistic target in the context of PTX-induced sensory neurotoxicity [17].

Against this background, Thymoquinone (TQ), the principal bioactive compound of Nigella sativa seeds [18], also referred to as 2-isopropyl-5-methyl-1,4-benzoquinone, has been reported to exhibit a wide range of potential pharmacological properties, including cardioprotective, hepatoprotective, nephroprotective, anticancer, anti-inflammatory, and osteoprotective effects [19,20]. Furthermore, TQ has gained increasing attention for its potential role in the management of neurodegenerative disorders such as Alzheimer’s and Parkinson’s disease [21,22,23]. Moreover, TQ has demonstrated significant neuroprotective effects in acute experimental models of CIPN by mitigating PTX-induced oxidative stress, inflammation, and cell death [24]. Since painful PIPN is a chronic condition that frequently persists long after treatment discontinuation, its long-term effects in chronic models remain largely unexplored. This study aimed to investigate both the effects of TQ in preventing PIPN *in vitro* while preserving its anticancer efficacy and to test TQ’s long-term effects in a chronic *in vivo* model of painful PIPN.

## 2. Materials and Methods

### 2.1. Drug Preparation

For the *in vitro* experiments, PTX powder (LC Laboratories, Woburn, MA, USA) was dissolved in absolute ethanol (EtOH) to prepare a 10 mM stock solution (stored at −20 °C), and subsequently diluted in culture media to achieve the desired working concentrations, resulting in a final EtOH concentration within a range previously established as non-toxic [25]. TQ Powder (Sigma Aldrich, Milan, Italy) was dissolved in absolute dimethyl sulfoxide (DMSO) to prepare a 200 mM stock solution (stored at −20 °C) and diluted in culture media to achieve the required concentrations.

For the *in vivo* experiments, PTX and TQ solutions were freshly prepared on the day of administration. PTX was dissolved in a vehicle solution consisting of 10% Tween-80, 10% EtOH, and 80% saline (NaCl 0.9%), at a dose of 10 mg/kg (1 mL/kg) and intravenously injected. TQ powder was dissolved in corn oil and orally administered at doses of 5 mg/kg and 10 mg/kg, at a concentration of 1 mL/kg.

### 2.2. In Vitro Experiments

#### 2.2.1. Evaluation of Potential Neuroprotective Effects of TQ Against PTX Neurotoxicity

All experimental procedures were evaluated and approved by the Ethics Committee for Animal Studies of the University of Milan Bicocca and by the Italian Ministry of Health (authorization number N. 93/2017-PR, notification number FB7CC.N.PWX).

As previously described [25], DRG from 15-day-old embryonic Sprague–Dawley rats were collected and cultured onto a single layer of rat-tail collagen coated in 35 mm dishes (4 ganglia/dish). The DRG were maintained in AN2 medium consisting of minimum essential medium MEM (Euroclone, Pero, Italy) supplemented with 10% Calf Bovine Serum (Euroclone, Pero, Italy), 1.4 mM L-Glutamine (Euroclone, Pero, Italy), 0.6% Glucose (Sigma-Aldrich, St. Louis, MO, USA), 10 ng/mL Nerve Growth Factor (NGF) (Thermo Fisher, Waltham, MA, USA) and fluorodeoxyuridine Fudr (10^−5^ M) to remove supporting cells. Cultures were incubated at 37 °C in a humidified atmosphere containing 5% CO_2_.

After 2 h, culture medium was removed and replaced with treatment medium containing PTX (50 nM), TQ (5 μM, 10 μM), or their combination. These concentrations were selected according to preliminary dose–response experiments. Untreated DRG represented the controls. After 24 h and 48 h of treatment, micrographs of DRG were taken under a light microscope Nikon Eclipse TS100 (TiEsseLab Srl, Milano, Italy), and the length of the longest neurite was measured with ImageJ software (version 1.54p, National Institutes of Health, Bethesda, MD, USA, https://imagej.net/ij/ accessed on 10 June 2026).

#### 2.2.2. Western Blotting

Total protein extracts were isolated from embryonic DRG explants following 24 h of treatment with each compound, using radio-immunoprecipitation assay (RIPA) lysis buffer. Proteins were subsequently resolved by electrophoresis on a graded 4–20% acrylamide SDS-PAGE gel and transferred onto a nitrocellulose membrane. The membrane was blocked with 5% non-fat milk in Tris-Buffered Saline containing 0.1% Tween-20 (TBS-T 0.1%) for 1 h at room temperature, followed by overnight incubation at 4 °C with the primary antibody anti-SIRT1 (cat. no. 9475S, rabbit, 1:1000; Cell Signalling Technology, Danvers, MA, USA). The membrane was then washed and incubated for 1 h at room temperature with the appropriate horseradish peroxidase (HRP)-conjugated anti-rabbit secondary antibody. Finally, immunoreactive protein bands were detected by enhanced chemiluminescence (ECL) and visualized using the Azure Biosystems 600 imaging system (Azure Biosystems, Dublin, CA, USA). Band intensities were subsequently quantified using Fiji (ImageJ, NIH, Bethesda, MD, USA) by densitometric analysis. Protein expression levels were normalised to β-actin as a loading control.

#### 2.2.3. Evaluation of the Potential Interfering Effect of TQ on PTX Antineoplastic Activity

An MTT assay was performed to evaluate whether TQ could interfere with the cytotoxic activity of PTX using the MCF-7 and MDA-MB231 human breast cancer cell lines (ATCC, Manassas, VA, USA). As previously described [26], MCF-7 cells were maintained in Roswell Park Memorial Institute medium (RPMI-1640) (Euroclone S.p.A., Milan, Italy) while MDA-MB-231 cells were cultured in Dulbecco’s Modified Eagle’s Medium (DMEM). Both media were supplemented with 1% L-glutamine, 1% penicillin/streptomycin (Euroclone, Pero, Italy), and 10% fetal bovine serum (FBS). Cells were maintained at 37 °C in a humidified incubator with 5% CO_2_ and passaged every 3–4 days upon reaching 80–90% confluency.

Cells were seeded at a density of 1 × 10^4^ cells/well in 96-well plates and treated with PTX (50 nM), TQ (5 µM or 10 µM), or their combination for 24 h or 48 h. At each time point, 100 µL of 3-(4,5-dimethylthiazol-2-yl)-2,5-diphenyltetrazolium bromide (MTT; 0.5 mg/mL; Sigma-Aldrich, USA) dissolved in phenol red-free DMEM was added to each well and incubated at 37 °C for 4 h. The MTT solution was then removed, and the resulting formazan crystals were dissolved in pure DMSO. Absorbance was measured at 570 nm using a microplate reader (BMG-Labtech, Ortenberg, Germany).

### 2.3. In Vivo Experiments

#### 2.3.1. Animal Ethics

All experimental procedures were performed in conformity with the institutional guidelines in compliance with national (D. L.vo 26/2014, Gazzetta Ufficiale della Repubblica Italiana, n.61, 14 March 2014) and international laws and policies (European Union directive 2010/63/UE; Guide for the Care and Use of Laboratory Animals, U.S. National Research Council, 1996). The experiments were evaluated and approved by the Italian Ministry of Health (Authorisation number N. 127/2024-PR).

#### 2.3.2. Animal Housing

Female Wistar rats weighing 175–200 g (at the beginning of the study) were purchased from Envigo Laboratory (Udine, Italy) and randomized into 12 animals/group. All rats were housed in controlled rooms under a 12 h light/dark schedule. They were maintained at 22 ± 2 °C with a relative humidity of 55 ± 10%. They were provided ad libitum access to food and water. To assess their general condition, rats were weighed twice weekly and observed daily for any signs of debilitation resulting from drug treatments. These signs included changes in appearance (i.e., kyphosis, rhinorrhea, mucosal dehydration, and piloerection), activity (decreased nesting and exploring), and behaviour (decreased eating, grooming, and drinking). Any animal exhibiting significant signs of distress or a body weight reduction of over 20% from the beginning of the study was thoroughly evaluated by an expert veterinarian in animal studies, who had the authority to withdraw from the study if necessary.

#### 2.3.3. Experimental Design

**Figure 1:**
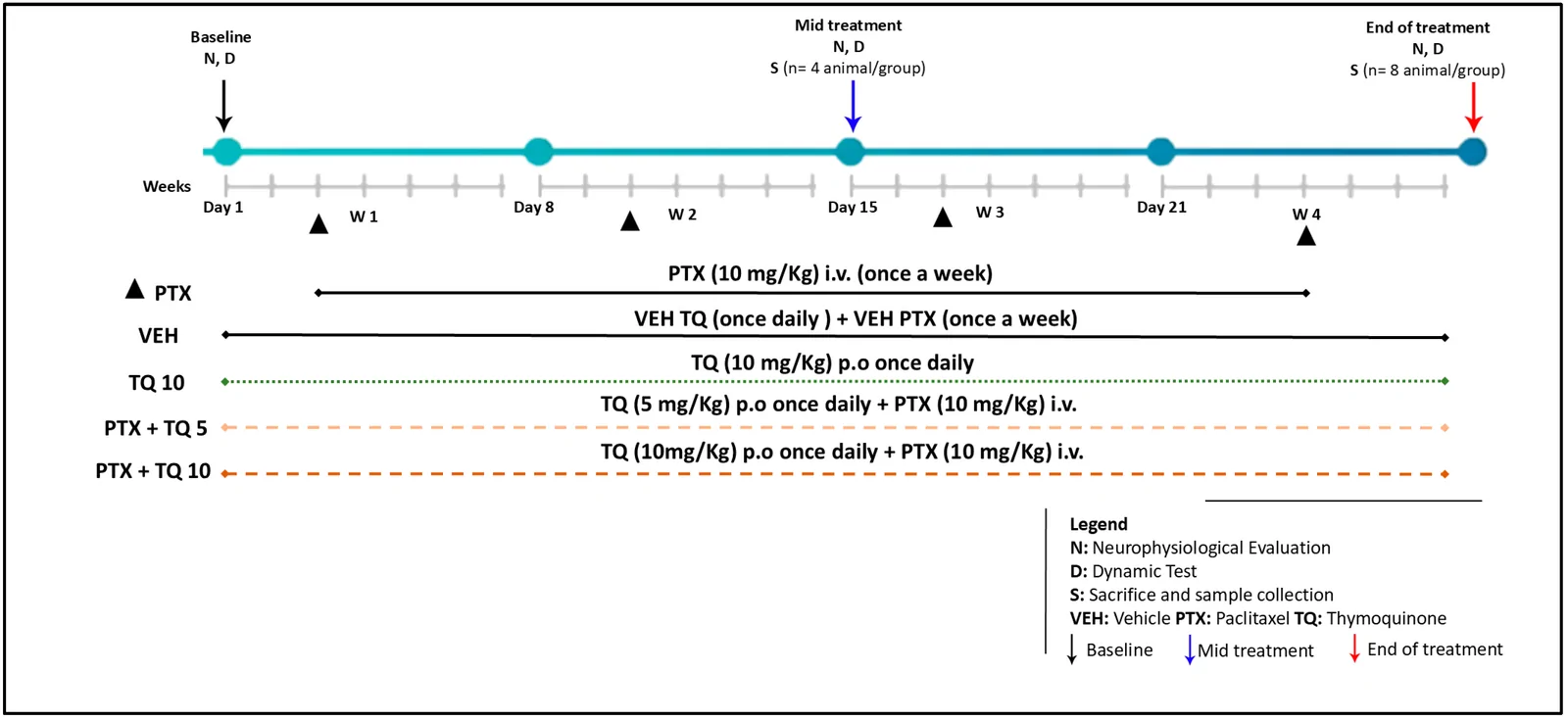
Schematic representation of study design.

To discern whether TQ could effectively prevent PTX-induced painful peripheral neuropathy, a preventive study was designed. Sixty female rats were assigned to 5 experimental groups (*n* = 12/group) based on baseline behavioral and neurophysiological responses. The number of animals per group (*n* = 12) was carefully selected in full compliance with the 3Rs reduction principle to ensure the production of reliable data and the acquisition of a sufficient number of biological samples for the intended analyses. Twelve rats received daily oral administrations of corn oil (1 mL/kg) for 4 weeks. Additionally, they received the PTX vehicle solution, consisting of 10% Tween-80, 10% EtOH absolute, and 80% saline solution, via i.v. administration once weekly for 4 weeks (VEH group). Twelve rats received PTX 10 mg/kg intravenously (i.v.) once a week for 4 weeks, resulting in a cumulative dose of 40 mg/kg (PTX group) [27]. Twelve rats were orally administered TQ (10 mg/kg) daily for 4 weeks, for a cumulative dose of 280 mg/kg (TQ group) [28,29]. The remaining 24 animals received PTX (10 mg/kg, i.v.) once weekly for 4 weeks, together with daily oral administration of TQ at 5 mg/Kg or 10 mg/Kg for 4 weeks (PTX + TQ 5 and PTX + TQ 10). On the day of PTX injection, TQ was injected 15 min before PTX (Figure 1) [30].

Throughout the experimental period, animal health status was monitored daily, and body weight was recorded twice a week during the treatment period to monitor general conditions. Neurophysiological studies (see Section 2.3.4) and behavioural tests (see Section 2.3.5) were performed at baseline, after 2 and 4 weeks of drug treatment for the evaluation of peripheral neuropathy and neuropathic pain development, respectively. After 2 weeks of drug treatment, 4 rats belonging to each group were sacrificed/euthanised for neuropathological (see Section 2.3.6 and Section 2.3.7) evaluations. The remaining rats continued until the study endpoint, after which they underwent final neurophysiological evaluations and behavioral tests before being sacrificed.

At both timepoints of sacrifice, animals were euthanized under deep anaesthesia induced by isoflurane overdose. Blood samples were collected from 4 rats/group at mid-treatment and from 8 rats/group at the end of treatment. These samples were analysed for hematological and biochemical parameters to evaluate the systemic toxicity of PTX and TQ. In addition, at each point, tissue samples including peripheral nerves, DRG and skin samples were harvested from 4 rats/groups for morphological and morphometrical neuropathological analysis.

#### 2.3.4. Assessment of Peripheral Neurotoxicity: Nerve Conduction Studies (NCS)

The onset and chronicization of PIPN were evaluated by measuring sensory nerve conduction velocity (SNCV) and sensory nerve action potential (SNAP) using the Matrix Light electromyography apparatus (Matrix Light, Micromed, Treviso, Italy) to assess peripheral sensory and motor nerve function. All procedures were executed under conditions of deep isoflurane anesthesia, with stringent maintenance of core body temperature at 37 ± 0.5 °C through the application of a heating pad. Assessments of the digital nerve were documented both at baseline and at the end of the treatment, while evaluations of the caudal nerve were conducted at mid-treatment and at the end of the study. Stainless steel needle electrodes (Ambu Neuroline; Ambu, Ballerup, Denmark) were utilized for the recordings, with strategic placement of both recording and stimulating electrodes for each nerve. The computation of SNCV was derived from the latency interval measured between the stimulus artifact and the initial response peak, accompanied by precise measurements of the distance separating the electrode sites. Additionally, SNAP was recorded, with the filtering settings established between 20 Hz and 3 kHz, and a sweep duration maintained at 0.5 ms [31].

#### 2.3.5. Assessment of Neuropathic Pain: Dynamic Aesthesiometer Test for Detection of Mechanical Allodynia

Pain sensitivity to mechanical stimuli was assessed using a dynamic plantar aesthesiometer apparatus (Ugo Basile Biological Instruments, Comerio, Italy), which gradually increased the mechanical force. At each timepoint, rats were positioned individually in plexiglass cages placed on a metal grid floor for a 15-min acclimation period. A pointed metallic filament (0.5 mm diameter) was then applied to the plantar surface of the hind paw, delivering a linearly increasing force that reached 50 g within 20 s. This stimulus prompted an evident voluntary hind paw withdrawal reaction/response, which was recorded to provide the mechanical nociceptive threshold and was registered six times, three times for each leg, to yield a mean value (expressed in grams). A cut-off time of 20 s was fixed to avoid damage [32].

#### 2.3.6. Assessment of Peripheral Neurotoxicity: Neuropathological Analyses

The caudal nerve (both proximal and distal segments) and L4–L6 dorsal root ganglia (DRG) were harvested from four animals in each group for morphological examination. As described in Ref. [32], nerve and DRG samples were promptly immersed in 4% paraformaldehyde/2% glutaraldehyde and 3% glutaraldehyde, respectively, and subsequently osmicated, dehydrated, and embedded in epoxy resin for light microscopy observations. Specifically, 1.5 µm-thick sections stained with toluidine blue were observed and scanned with a Nexcope Ne920 AUTO light microscope (TiEsseLab, Milano, Italy) at a magnification of 40X (for caudal nerves) or 20X for DRG.

#### 2.3.7. Intra-Epidermal Nerve Fiber Density (IENF)

IENF density was quantified in the hind paw footpad in three animals/group to examine eventual damage of small unmyelinated peripheral nerve fibers. This parameter is consequently correlated with neuropathic pain [33]. As previously described in [34], skin biopsies were collected from 3 animals/group/timepoint and immediately fixed in 2% PLP (paraformaldehyde–lysine–sodium periodate) solution for 24 h at 4 °C, cryoprotected, and serially sectioned with a cryostat to obtain 20-µm-thick longitudinal sections. Three sections from each sample were randomly selected and subjected to immunostaining with a primary rabbit antibody against PGP 9.5 (GeneTex, Irvine, CA, USA) in combination with biotinylated anti-rabbit IgG and Vector SG substrate kit peroxidase (Vector Laboratories, Burlingame, CA, USA) using a free-floating protocol. Only the nerve fibers crossing the dermal/epidermal junction were quantified; the length of the epidermis was recorded, and the IENF density was calculated as the ratio of the number of positive IENF to the measured epidermal length (IENF/mm).

### 2.4. Statistical Analysis

All data were analysed using GraphPad Prism 8 software (San Diego, CA, USA). Data distribution was assessed using the Shapiro–Wilk normality test before statistical analysis. For *in vitro* experiments, data are expressed as mean ± standard deviation (SD), and group differences were evaluated using one-way ANOVA followed by Tukey’s post-hoc test for pairwise comparisons.

For *in vivo* data, body weight data are expressed as mean ± SD and were analysed using a two-way ANOVA with a mixed-effects model, with treatment and time as fixed factors, followed by Bonferroni-adjusted multiple comparisons.

For all remaining *in vivo* endpoints, including behavioural data, neurophysiological parameters, IENF density, and blood parameters, violations of the normality assumption were detected in one or more groups and/or timepoints; data are therefore expressed as median (IQR) and were analysed using the Kruskal–Wallis test followed by Dunn’s multiple-comparisons test, performed separately at each timepoint. A *p*-value < 0.05 was considered statistically significant.

## 3. Results

### 3.1. In Vitro Results

#### 3.1.1. TQ Transiently Protects Against PTX-Induced Neurotoxicity in *In Vitro* DRG

**Figure 2:**
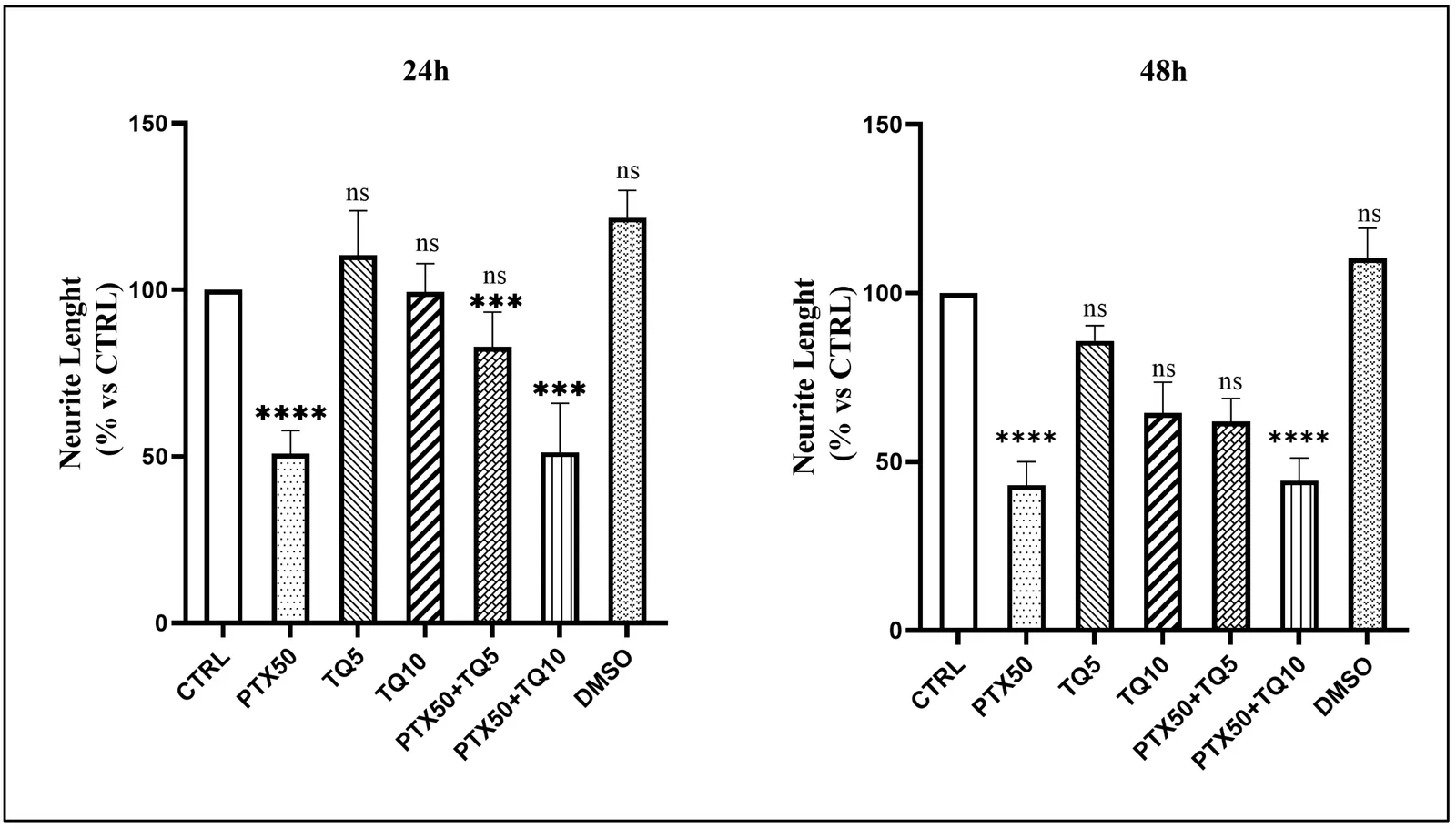
Neurite outgrowth from DRG treated with PTX, TQ or their combination for 24 and 48 h. Graphs represent neurite outgrowth as a percentage with respect to the CTRL group (**** *p* < 0.0001 vs. CTRL; *** *p* < 0.001 vs. PTX; ^ns^ *p* > 0.05). See Section 2 for abbreviations.

To characterize the effect of TQ against PTX-induced neurotoxicity *in vitro*, we used the well-established embryonic DRG explant model. In this model, neurite elongation serves as the primary parameter for assessing both neurotoxic and neuroprotective effects [25]. At both timepoints, TQ alone, at either concentration, did not significantly alter neurite length relative to the CTRL group (*p* > 0.05), confirming the absence of intrinsic neurotoxicity at the tested doses. Nevertheless, as expected, exposure to PTX (50 nM) induced a marked and significant reduction in neurite length at both 24 h and 48 h (**** *p* < 0.0001 vs. CTRL; Figure 2). In contrast, the co-treatment with PTX + TQ5 significantly attenuated this neurite impairment at 24 h, compared to PTX alone (*** *p* < 0.001 vs. PTX; Figure 2), demonstrating a transient neuroprotective effect at the lower dose. However, this protective effect was not sustained at 48 h. Furthermore, the co-treatment with PTX + TQ10 failed to rescue neurite elongation at either timepoint.

#### 3.1.2. TQ Prevents PTX-Induced SIRT1 Downregulation in Embryonic DRG Neurons

**Figure 3:**
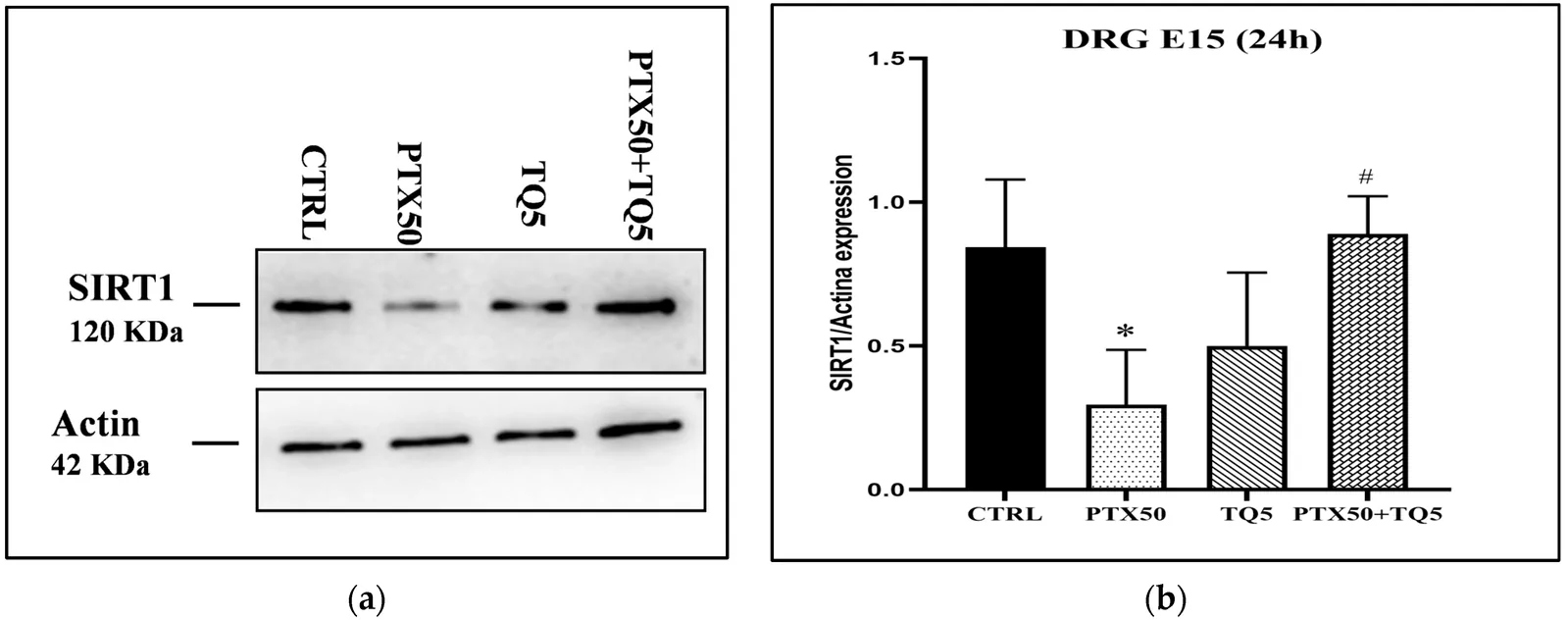
Effect of TQ on PTX-induced SIRT1 downregulation in embryo DRG explant after 24 h of treatment. (**a**) Representative Western blot image of SIRT1 expression. (**b**) Comparative levels of SIRT1 expression in embryo DRG explant normalised to levels of β-actin expression (*n* = 3). (* *p* < 0.05 vs. CTRL and # *p* < 0.05 vs. PTX). The original western blots are provided in Supplemental Figure S1, See Section 2 for abbreviations.

To elucidate the molecular mechanisms underlying the TQ-mediated effect *in vitro*, SIRT1 protein expression was assessed by western blot in embryonic DRG explants after 24 h of treatment with PTX, TQ, or their combination. This timepoint was selected based on the neurite outgrowth results, which identified 24 h as the window of TQ cytoprotective activity. As shown in Figure 3, PTX treatment induced a significant downregulation of SIRT1 protein expression in embryonic DRG explants compared to the CTRL (*p* = 0.019), indicating that PTX-induced neurotoxicity in sensory neurons is associated with suppression of SIRT1 signaling. However, co-treatment with PTX + TQ prevented this downregulation (*p* = 0.018 vs. PTX and *p* > 0.05 vs. CTRL), suggesting a potential association between SIRT1 preservation and the neuroprotective effect of TQ observed at this timepoint (Figure 3).

#### 3.1.3. TQ Does Not Interfere with PTX-Induced Antineoplastic Effect

We further investigated TQ at its effective, non-neurotoxic concentration, as evidenced by the absence of significant effects on neurite length in embryonic DRG explants, to assess whether it interferes with the cytotoxic efficacy of PTX against MCF-7 and MDA-MB231 breast cancer cells, using the MTT assay at 24 h and 48 h.

**Figure 4:**
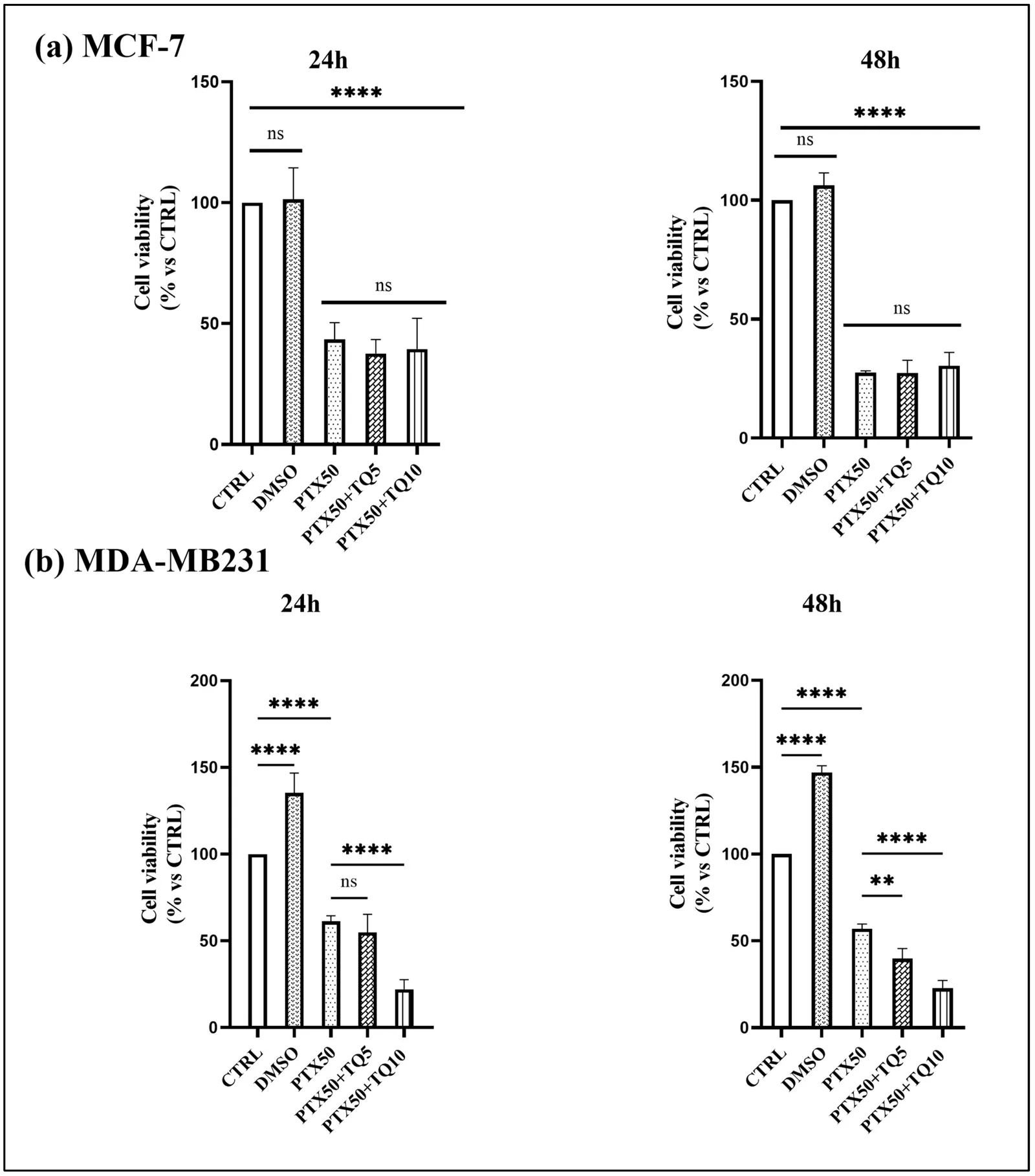
Effect of TQ and PTX co-treatment for 24 h and 48 h on the viability of MCF-7 (**a**) and MDA-MB-231 (**b**) breast cancer cell lines. Graphs represent cell viability as a percentage relative to the CTRL group (**** *p* < 0.0001; ** *p* < 0.01; ^ns^ *p* > 0.05). See Section 2 for abbreviations.

In MCF-7 cells, PTX (50 nM) induced a significant reduction in cell viability at both timepoints (*p* < 0.0001 vs. CTRL; Figure 4a), which was not significantly altered by co-treatment with TQ at either concentration (*p* > 0.05; Figure 4a). Similarly, PTX significantly decreased cell viability in MDA-MB-231 cells at both time points. Notably, co-treatment with TQ10 significantly potentiated PTX-induced cytotoxicity at both 24 and 48 h (*p* < 0.0001; Figure 4b), whereas TQ5 produced an additional cytotoxic effect at 48 h (*p* < 0.001; Figure 4b). Collectively, these findings demonstrate that TQ does not compromise the antineoplastic activity of PTX and may further enhance its cytotoxic efficacy against breast cancer cells.

### 3.2. In Vivo Results

#### 3.2.1. TQ Does Not Show Any General Toxicity *In Vivo*

Throughout the experiment, the daily oral administration of TQ and the weekly intravenous administration of PTX, whether given alone or in combination, were well tolerated by the animals. They continued to groom, explore their surroundings, and climb on their wire cages during the course of drug treatments.

**Figure 5:**
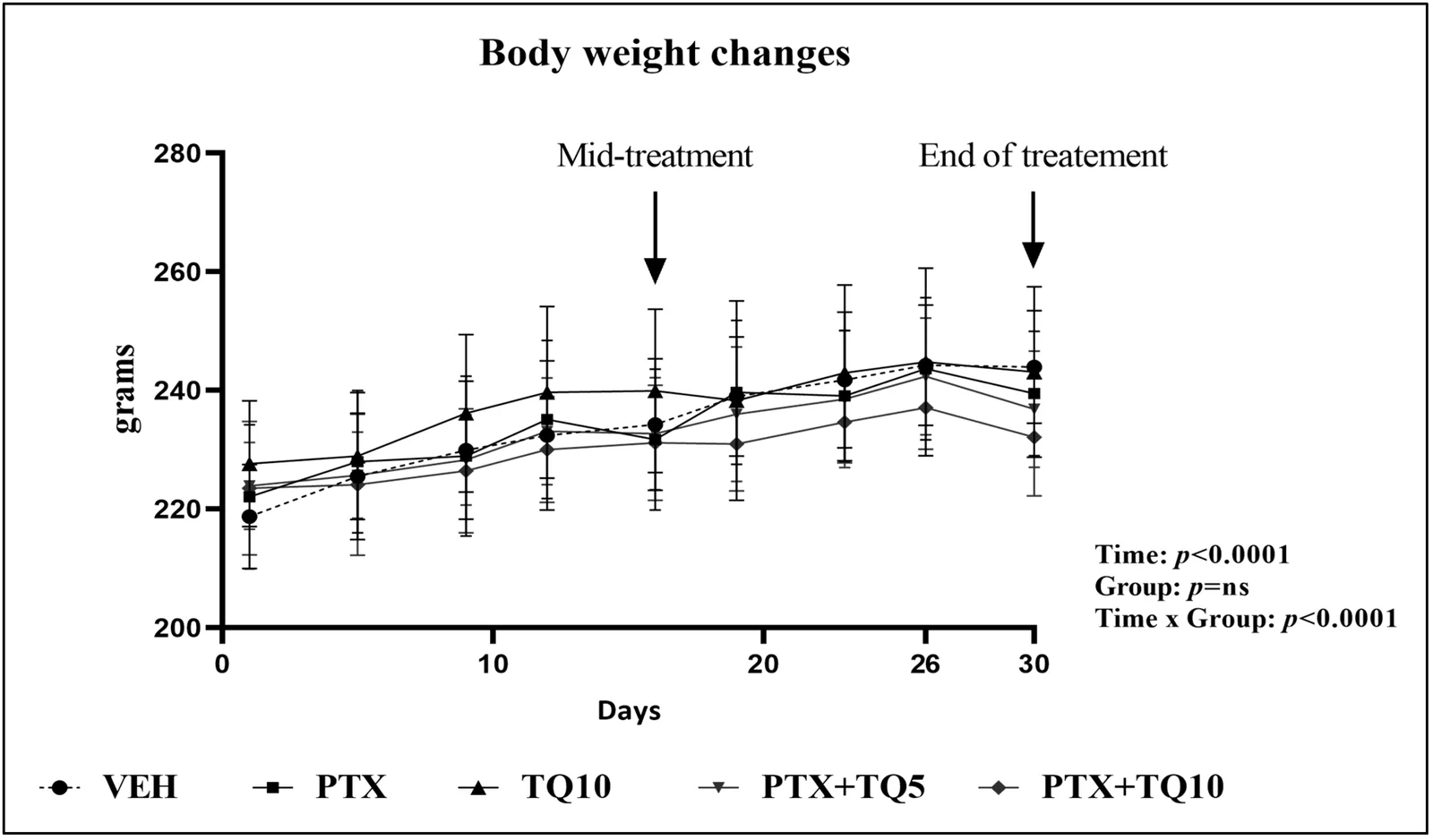
Body weight changes during the study period. The body weights (mean ± SD) of each group of rats were recorded twice weekly (*n* = 12/group during the mid-treatment period, *n* = 8/group until the end of treatment).

A highly significant increase in body weight over time was observed within groups (*p* < 0.0001) due to the physiological growth of the animals; however, no significant differences were found between groups during the experiment (*p* > 0.05), indicating that neither PTX nor TQ, alone or combined, significantly altered body weight trajectory compared to the VEH group (Figure 5).

#### 3.2.2. Hematological and Biochemical Parameters: Evidence of Mild Erythroid Myelosuppression

**Figure 6:**
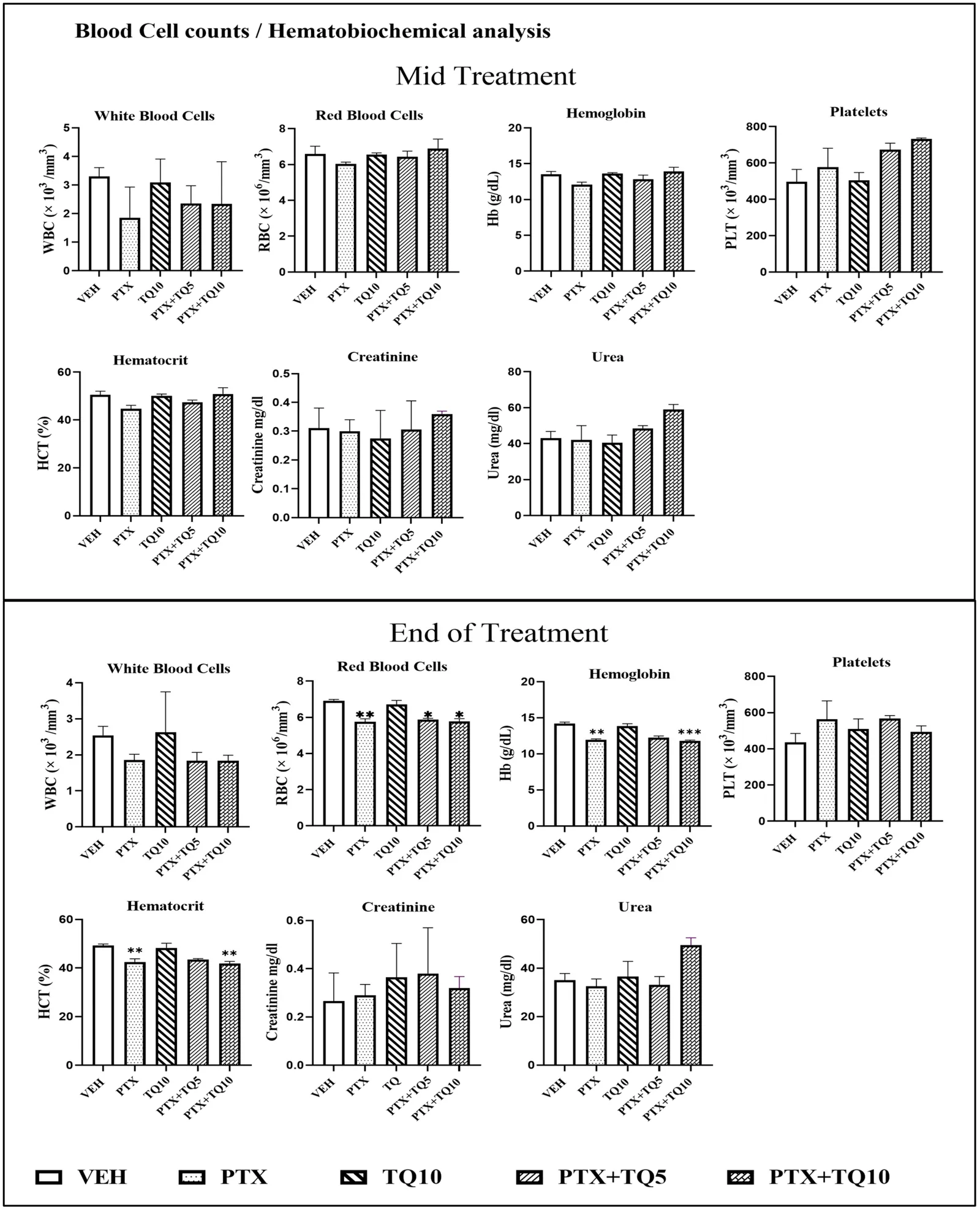
Hematological and biochemical analysis at mid- and end-of-treatment. Data are expressed as median (IQR). *n* = 4 rats/group at mid-treatment; *n* = 8 rats/group at end of treatment. * *p* < 0.05, ** *p* < 0.01, *** *p* < 0.001 vs. VEH. See Section 2 for abbreviations.

Haematological and biochemical parameters were assessed at mid- and end-of-treatment timepoints (weeks 2 and 4) to monitor the systemic tolerability of PTX and TQ administration across all experimental groups (Figure 6). At both time points, the administration of TQ alone was not associated with any significant alterations in the measured hematological or biochemical parameters, including white blood cells (WBC), red blood cells (RBC), hemoglobin (Hb), hematocrit (HCT), platelets (PLT), creatinine, and urea compared to the VEH group. These results indicate a lack of systemic toxicity associated with TQ administration. At mid-treatment, the administration of PTX, either alone or in combination with TQ, similarly showed no significant effect on any of the evaluated hematological or biochemical parameters compared to VEH groups (*p* > 0.05). However, by the end of the treatment, animals treated with PTX alone or in combination with TQ10 exhibited a significant reduction in RBC counts (PTX: *p* = 0.002, PTX + TQ5: *p* = 0.032, PTX + TQ10: *p* = 0.012), Hb levels (PTX: *p* = 0.003, PTX + TQ10: *p* = 0.002), and HCT (PTX: *p* = 0.006, PTX + TQ10: *p* = 0.001), compared to the VEH group. Despite these reductions in erythroid parameters, all values remained above the established physiological thresholds for this species, suggesting mild erythroid myelosuppression [35,36]. Furthermore, at the end of treatment, PTX and TQ administration, alone or in combination, did not affect WBC counts, platelet levels, or renal function markers at either timepoint, indicating preserved leukocyte and thrombocyte lineages and the absence of nephrotoxicity.

#### 3.2.3. TQ Totally Prevents PTX-Induced Mechanical Allodynia, and Transiently Protects Against PTX-Induced Small Fiber Loss in Skin Biopsies

**Figure 7:**
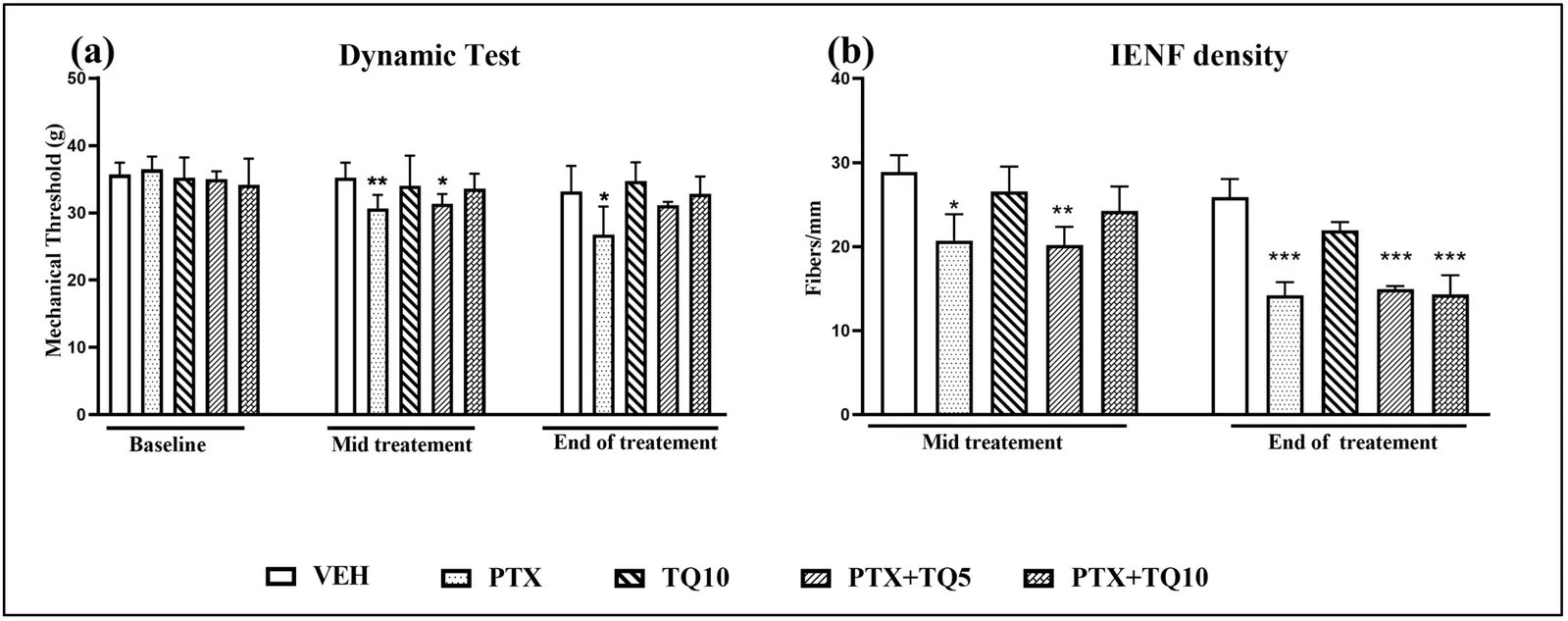
Effects of PTX and TQ on mechanical allodynia and skin IENF density. (**a**) Dynamic Plantar Aesthesiometer test (*n* = 12 rats/group at baseline and mid-treatment; *n* = 8 rats/group at end of treatment). (**b**) Skin IENF density (*n* = 3 rats/group at mid-treatment and end of treatment). Data are expressed as median (IQR). * *p* < 0.05, ** *p* < 0.01, *** *p* < 0.001 vs. VEH. See Section 2 for abbreviations.

Mechanical withdrawal threshold, a key behavioural indicator of mechanical allodynia and a hallmark of peripheral neuropathic pain, was assessed longitudinally across all experimental groups at mid- and end-of-treatment. At mid-treatment, PTX and PTX + TQ5 groups exhibited a statistically significant reduction in mechanical threshold compared to the VEH group (*p* = 0.0052 and *p* = 0.01, respectively; Figure 7a), indicating the development of mechanical allodynia. In contrast, no significant difference was observed in TQ (10 mg/kg) alone or in combination with PTX compared to the VEH group (*p* > 0.05; Figure 7a), suggesting that TQ10 was able to prevent the mechanical allodynia induced by PTX. At the end of treatment, both TQ5 and TQ10 groups were able to prevent PTX-induced mechanical allodynia (*p* > 0.05, Figure 7a).

The quantitative analysis of skin IENF further substantiated these findings. As expected, PTX caused a significant reduction in IENF density. Notably, this fiber loss was prevented by TQ10 only at mid-treatment (*p* > 0.05, Figure 7b), but not at the end of treatment.

#### 3.2.4. TQ Does Not Prevent PTX-Induced Neurophysiological Impairment or the Distal-to-Proximal Progression of Caudal Nerve Degeneration

**Figure 8:**
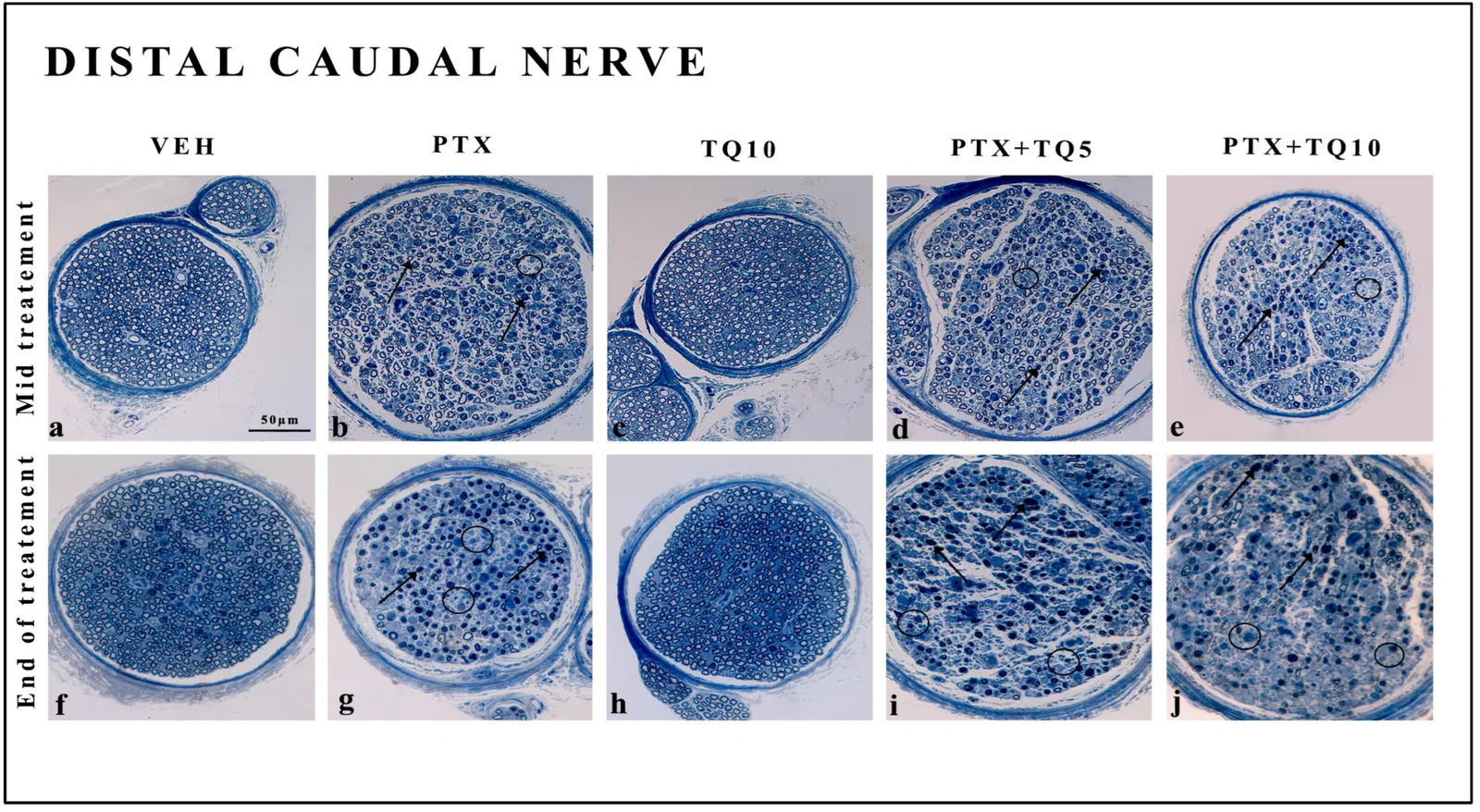
Representative images of distal caudal nerve samples at mid-treatment (**a**–**e**) and the end of treatment (**f**–**j**). Circles and arrows represent fiber loss and degeneration, respectively.

**Figure 9:**
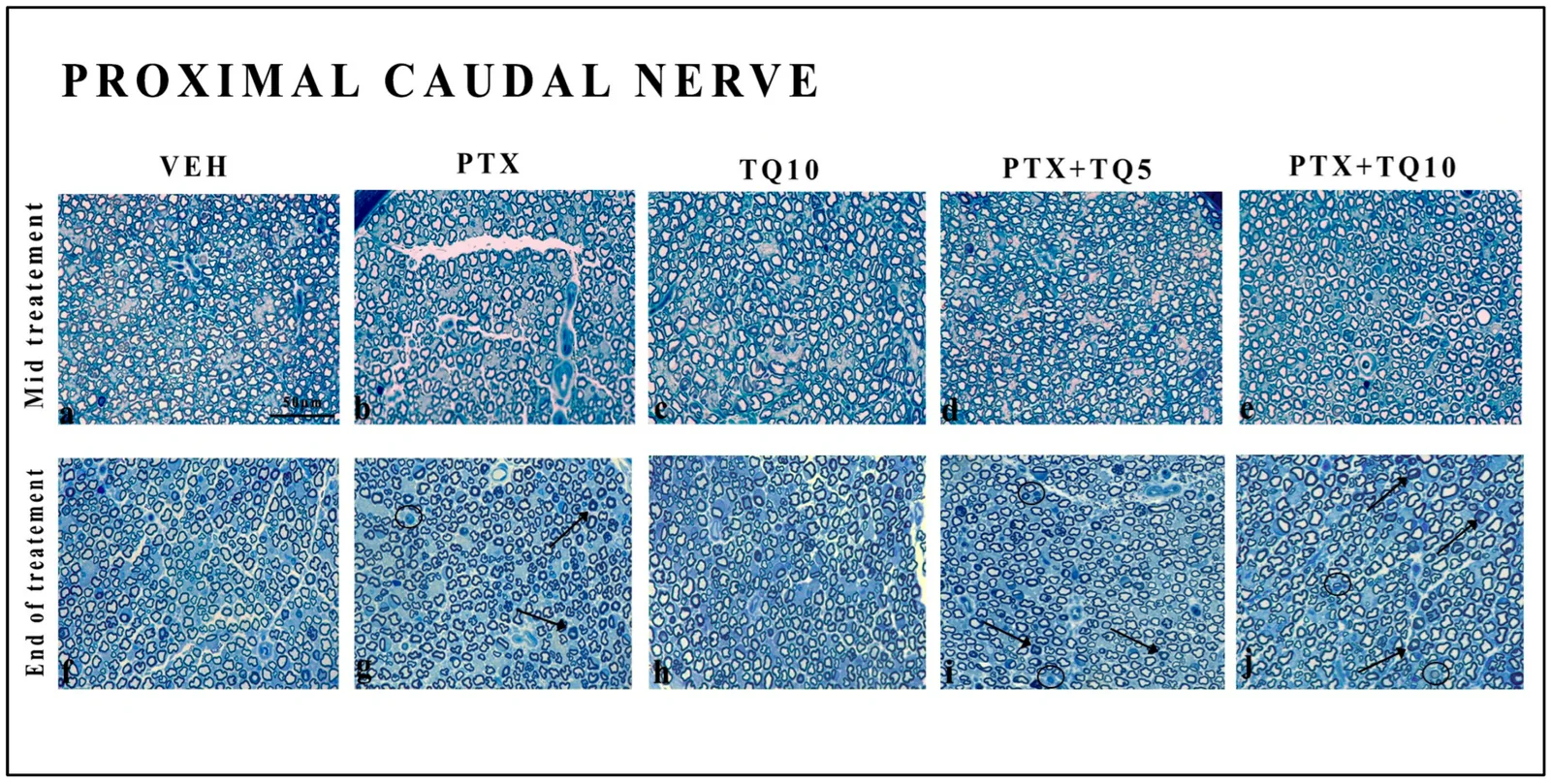
Representative images of proximal caudal nerve samples at mid-treatment (**a**–**e**) and at the end of treatment (**f**–**j**). Circles and arrows represent fiber loss and degeneration, respectively.

To evaluate the effects of chronic PTX exposure and TQ co-administration on large myelinated fibers, SNAP amplitude and SNCV were measured in both the digital and caudal nerves. The results revealed no significant differences in digital nerve SNAP amplitude or SNCV across all groups, while caudal nerve recordings showed progressive reductions in both parameters across all PTX-containing groups by the end of treatment. However, no neurotoxic effect of TQ alone was detected (Supplemental Figure S2). Morphological examination of the caudal nerve corroborated these neurophysiological findings, confirming a time-dependent and distal-to-proximal degeneration pattern typical of PIPN [37]. In distal segments, degeneration of myelinated fibres and fiber loss were already evident in all PTX-treated groups at mid-treatment compared to the VEH group (Figure 8b,d,e), worsening markedly by the end of treatment (Figure 8g,i,j). In contrast, the proximal caudal nerves exhibited structural integrity at mid-treatment, with mild morphological changes characterised by degeneration of myelinated fibres and fiber loss by the end of treatment (Figure 9g,i,j). Collectively, these findings demonstrate that TQ co-administration failed to prevent PTX-induced neurophysiological impairment or peripheral nerve structural degeneration at either dose or time point.

## 4. Discussion

PIPN is a prevalent and debilitating complication of PTX-based chemotherapy, often leading to dose reduction or treatment cessation, and no preventive neuroprotective strategy is currently recommended for its management [38,39]. As we previously summarized [24], several preclinical studies have reported the neuroprotective effects of TQ against chemotherapy-induced neurotoxicity. However, most of these investigations were conducted in acute or subacute models, which do not fully represent the cumulative neurotoxic burden that can arise from clinical chemotherapy regimens. The present study is among the first to evaluate TQ in a chronic model of chemotherapy-induced peripheral neuropathy, combining both *in vitro* and *in vivo* experimental approaches.

We first assessed the neuroprotective effects of TQ against PTX-induced neurotoxicity using the embryonic DRG explant model, a well-established experimental model to evaluate chemotherapy-induced neurotoxicity [25,40]. Our findings demonstrated that PTX induced significant neurotoxicity after both 24 h and 48 h of exposure, which was significantly prevented by TQ5 at 24 h. To date, no study has evaluated the neuroprotective potential of TQ in an embryonic DRG explant model. Interestingly, Üstün et al. (2018) [41] reported a complete, dose-dependent neuroprotective effect of TQ against cisplatin-induced neurotoxicity in adult mouse DRG primary cultures, promoting both neuronal cell viability and neurite outgrowth. The discrepancy between their findings and the current results may be elucidated by the distinct molecular and cellular mechanisms through which cisplatin and PTX induce peripheral neurotoxicity. Cisplatin primarily acts through DNA adduct formation, triggering apoptotic and necrotic cell death in sensory neurons [42], against which TQ’s well-documented anti-apoptotic and cytoprotective properties may provide more effective neuroprotection [43,44]. In contrast, PTX stabilizes microtubules and impairs axonal transport, leading to a non-apoptotic dying-back axonopathy rather than neuronal cell body loss [45]. This mechanism may be less amenable to the anti-apoptotic effects of TQ and could contribute to the more limited neuroprotection observed in the present model.

SIRT1 is a NAD+-dependent histone deacetylase belonging to the sirtuin family, known to regulate a wide range of cellular processes, including metabolism, stress resistance, and cell survival [46,47]. Many studies have reported that SIRT1 plays a critical role in neuronal survival and axonal integrity, promoting neurite outgrowth through mTOR signaling inhibition [48,49]. Of particular relevance, SIRT1 has been implicated in the pathogenesis of CIPN, given that nicotinamide riboside, a NAD+ precursor and indirect SIRT1 activator, has been reported to attenuate PIPN, highlighting SIRT1 as a promising molecular target for neuroprotective intervention [17,50]. In line with this, our results revealed that PTX significantly downregulated SIRT1 protein expression in embryonic DRG neurons and TQ significantly prevented PTX-induced downregulation at 24 h, suggesting a potential association between SIRT1 preservation and the neuroprotective effect of TQ observed at this timepoint.

The mechanism underlying PTX-induced SIRT1 downregulation may relate to PTX’s well-documented ability to induce oxidative DNA damage and NF-κB-mediated neuroinflammation in sensory neurons. NF-κB activation and oxidative DNA damage are well-known inducers of cellular stress that lead to the transcriptional downregulation of SIRT1 (often mediated by microRNAs or NAD+ depletion driven by PARP hyperactivation consequent to DNA damage). Indeed, in other PTX-induced neurotoxicity models, SIRT1 downregulation has been associated with NF-κB activation and increased oxidative stress markers, including NADPH oxidase 2 (NOX2) and NADPH oxidase 4 (NOX4) [50,51]. In this context, the well-established antioxidant and anti-inflammatory properties of TQ may play a protective role in preserving SIRT1 expression by mitigating oxidative damage and neuroinflammatory signaling induced by PTX. This is further supported by earlier reports indicating that TQ directly activates SIRT1 through AMPK-dependent mechanisms in neuroinflammatory models [52].

To better characterize the clinical relevance of TQ as a potential adjuvant, we evaluated whether TQ co-administration interferes with the cytotoxic efficacy of PTX against MCF-7 and MDA-MB231 breast cancer cells. TQ at the non-neurotoxic concentrations tested did not significantly alter PTX cytotoxicity in MCF-7 cells at either time point, confirming the absence of interference with PTX anticancer activity. These findings corroborate those of Xing et al. (2023) [53], who similarly reported that TQ did not compromise PTX cytotoxicity in the same breast cancer cell line. Furthermore, in MDA-MB-231 triple-negative breast cancer cells, TQ significantly potentiated PTX cytotoxicity at both 24 h and 48 h, consistent with the synergistic interaction reported by Khazaei et al. (2024) [54]. Collectively, these findings demonstrate that TQ does not compromise the antineoplastic activity of PTX and may enhance its cytotoxic efficacy in an aggressive breast cancer cell model.

In addition, we investigated the neuroprotective effects of TQ in a well-established chronic PIPN rat model using a multimodal approach combining behavioural, neurophysiological, and histological assessments at mid- and at the end of PTX treatment [32]. The main findings of the *in vivo* part of this study are that (1) the chronic PIPN model employed was characterised by progressive mechanical allodynia, loss of IENF density, electrophysiological dysfunction, and peripheral nerve axonopathy, which are consistent with the clinical features of PIPN observed in patients [55]; (2) TQ consistently prevented PTX-induced mechanical allodynia throughout the 4-week treatment period; (3) TQ transiently attenuated IENF depletion, but (4) TQ failed to prevent neurophysiological and morphological alterations in peripheral nerves induced by chronic PTX exposure.

TQ’s consistent prevention of mechanical allodynia is in line with the results of Xing et al. (2023) [53], who demonstrated that TQ alleviates PIPN by suppressing the TLR4-MyD88 inflammatory pathway in an acute PIPN model. Moreover, similar anti-nociceptive effects of TQ have been reported in vincristine-induced neuropathy models, where TQ attenuated mechanical allodynia and thermal hyperalgesia through modulation of oxidative stress and neuroinflammatory pathways [56]. Notably, despite the robust analgesic effect observed, TQ co-administration only transiently preserved IENF density at mid-treatment, with complete small-fibre depletion observed across all PTX groups by the end of treatment. This dissociation between persistent analgesic efficacy and structural small fibre loss has been similarly reported for other neuroprotective candidates in PIPN models [57], suggesting that analgesic compounds may modulate pain signalling independently of structural nerve fibre preservation under prolonged chemotherapy exposure.

Furthermore, the neuroprotective effect of TQ was not associated with improvements in caudal SNCV or SNAP amplitude, suggesting that its protective action was insufficient to preserve the integrity of large myelinated fibres. These findings were corroborated by histological analyses, which revealed severe axonopathy in all PTX-treated groups at both time points. This lack of structural neuroprotection is not unexpected, given the early onset, severe and progressive nature of PTX-induced axonal degeneration, also previously described [32], suggesting that the cumulative neurotoxic burden of the chronic four-week PTX protocol likely exceeded TQ’s neuroprotective capacity at the doses tested. Collectively, these findings indicate that under chronic PTX exposure, TQ primarily acts as an analgesic agent rather than a structural neuroprotectant in the context of chronic PIPN.

The inverse dose–response observed both *in vitro* and *in vivo* may be compatible with a hormetic mechanism, a biphasic dose–response phenomenon increasingly documented for phytochemicals, including TQ [58]. It should be noted that the doses used *in vitro* (5 and 10 µM) and *in vivo* (5 and 10 mg/kg) were selected independently and do not directly correspond to one another, as extrapolation between *in vitro* µM concentrations and systemic mg/kg doses is pharmacologically unwarranted given the complex determinants governing drug exposure at peripheral nerve tissue *in vivo*, including absorption, protein binding, metabolic transformation, and blood–nerve barrier permeability. Elucidating this exposure–response relationship would require a pharmacometrics approach that integrates pharmacokinetic and pharmacodynamic modelling to define the optimal dosing regimen that achieves therapeutically relevant TQ concentrations at peripheral nerve targets. In parallel, enhanced drug delivery strategies such as nanostructured lipid carriers represent a promising avenue to improve TQ bioavailability and, consequently, its structural neuroprotective efficacy in future chronic PIPN studies.

Several limitations of the present study warrant consideration. These include the absence of *in vivo* mechanistic assessments and empirical sample size determination. With respect to the animal model, experiments were conducted exclusively in female Wistar rats, a choice driven by the clinical relevance of the model, given that PTX-based regimens are predominantly administered in the treatment of breast and ovarian cancers, and to ensure comparability with our group’s previous studies conducted under the same experimental conditions [32]. Given the mounting evidence that biological sex plays a significant role in modulating the phenotype of PIPN and its underlying mechanisms in preclinical models [59,60], incorporating male animals in future investigations is crucial for fully realizing the translational implications of these results.

## 5. Conclusions

The present study offers the first preclinical characterisation of TQ’s effects in a chronic PIPN model, demonstrating consistent attenuation of PTX-induced mechanical allodynia without compromising PTX antineoplastic activity in breast cancer cell lines. However, TQ did not prevent structural peripheral nerve degeneration under chronic PTX exposure, indicating that its primary *in vivo* action is analgesic rather than neuroprotective. Collectively, these findings underscore the necessity of future investigations integrating *in vivo* mechanistic endpoints, pharmacokinetic modelling, and advanced drug delivery strategies to establish the translational potential of TQ as a candidate preventive strategy for chronic CIPN.

## Data Availability

The original contributions presented in this study are included in the article/Supplementary Materials. Further inquiries can be directed to the corresponding authors.

## Supplementary Materials

The following supporting information can be downloaded at: https://www.mdpi.com/article/10.3390/biom16091282/s1, Figure S1: Original Western Blot images for SIRT1 and Actin; Figure S2: Effects of TQ on sensory nerve conduction velocity (SNCV) and sensory nerve action potential (SNAP) amplitude.
