# Supplementary material for: Thymoquinone Is Effective in Painful Paclitaxel-Induced Peripheral Neuropathy Without Compromising Anticancer Activity
Source: Biomolecules. 2026 Sep 4;16(9):1282. doi: 10.3390/biom16091282 (PMC13604513; doi:10.3390/biom16091282)
Supplement: Supplementary file 1 [file biomolecules-16-01282-s001.zip › biomolecules-4439790-supplementary.pdf]

Supplementary Figures

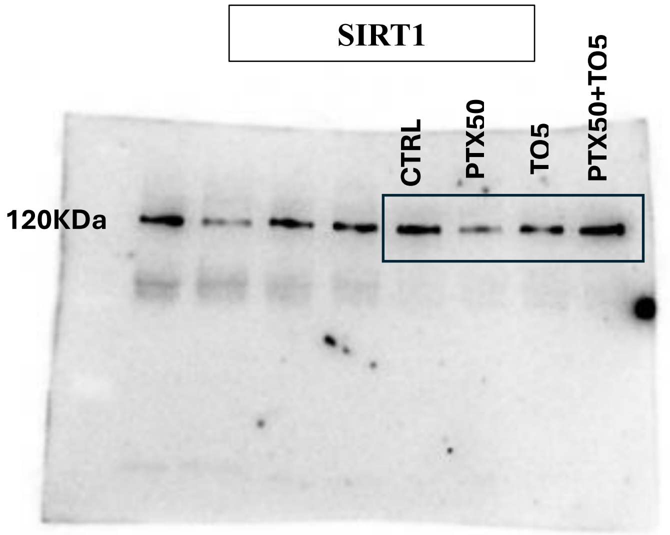

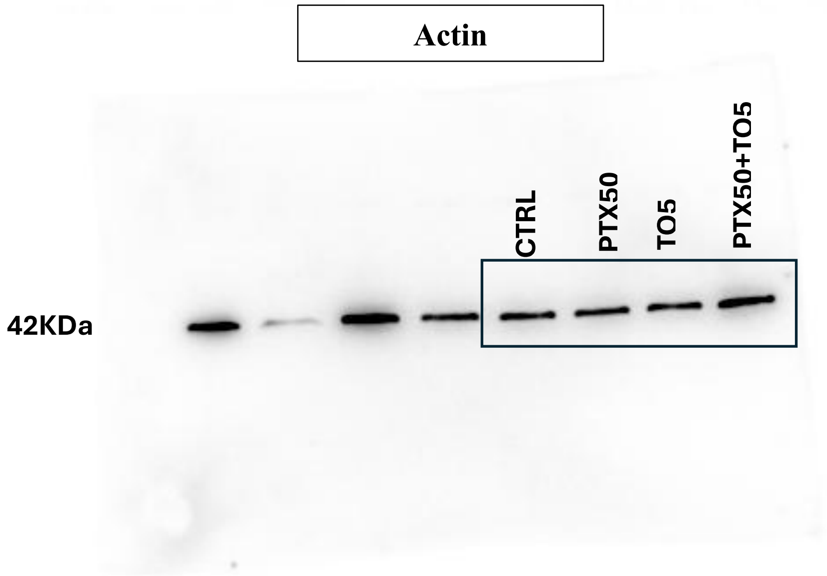

Figure S1: Original Western Blot images for SIRT1 and Actin. The selected lanes are indicated by the rectangle.

Nerve conduction studies

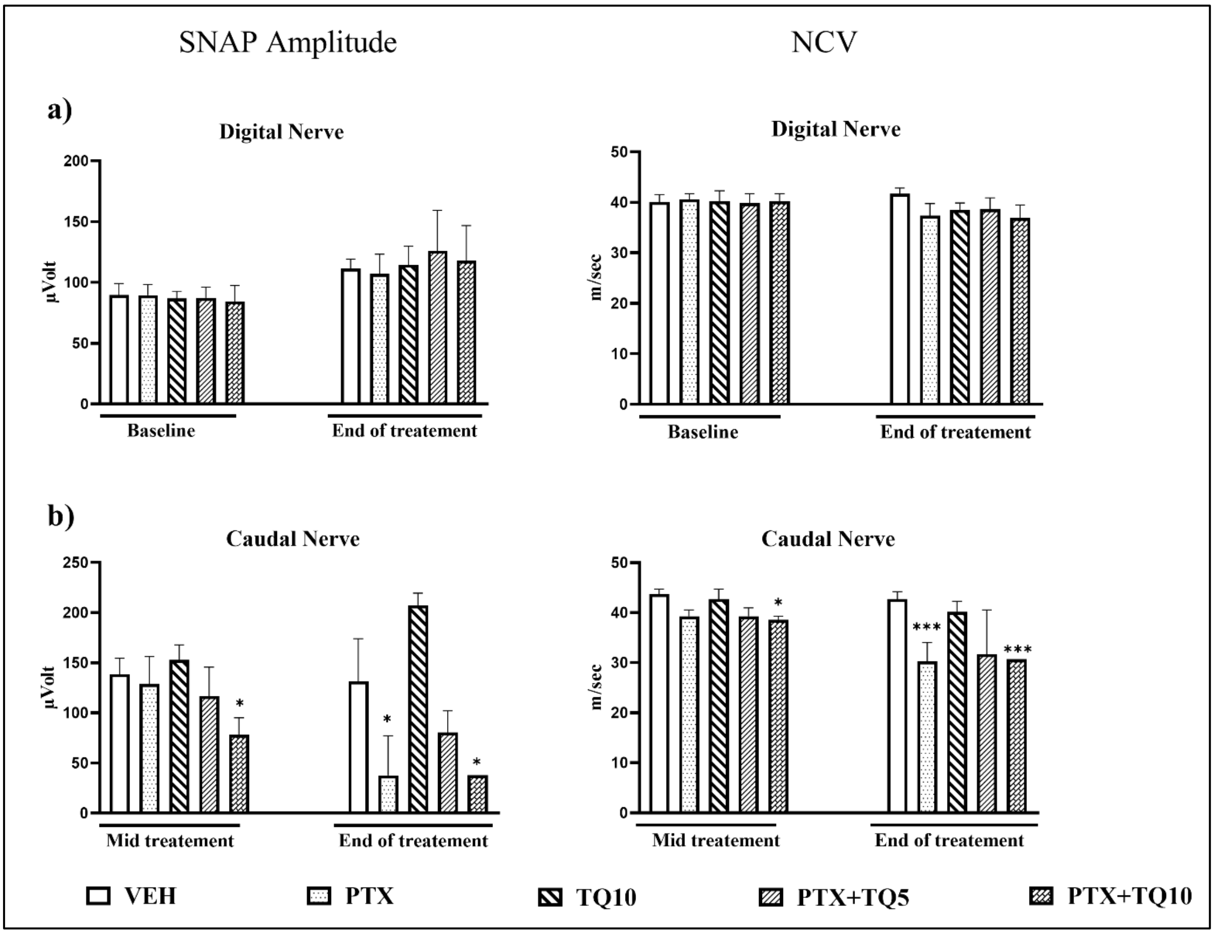

Figure S2: Effects of TQ on sensory nerve conduction velocity (SNCV) and sensory nerve action potential (SNAP) amplitude. (a) Digital nerve SNCV and SNAP amplitude. (b) Caudal nerve SNCV and SNAP amplitude. *$p < 0.05$, ***$p < 0.001$ vs. VEH. Data are expressed as median (IQR). n = 12 rats/group at baseline and mid-treatment; n = 8 rats/group at end of treatment.
